# A phase-I study of lapatinib in combination with foretinib, a c-MET, AXL and vascular endothelial growth factor receptor inhibitor, in human epidermal growth factor receptor 2 (HER-2)-positive metastatic breast cancer

**DOI:** 10.1186/s13058-017-0836-3

**Published:** 2017-05-02

**Authors:** Stephen K. Chia, Susan L. Ellard, Mihaela Mates, Stephen Welch, Catalin Mihalcioiu, Wilson H. Miller, Karen Gelmon, Caroline Lohrisch, Vikaash Kumar, Sara Taylor, Linda Hagerman, Rachel Goodwin, Tao Wang, Shingo Sakashita, Ming S. Tsao, Elizabeth Eisenhauer, Penelope Bradbury

**Affiliations:** 1 Medical Oncology, British Columbia Cancer Agency (BCCA), Vancouver, BC Canada; 2 Medical Oncology, BCCA, Kelowna, BC Canada; 3 Queen’s University and Cancer Centre of South Eastern Ontario at Kingston General Hospital, Kingston, ON Canada; 4 London Regional Cancer Program, London, ON Canada; 5 Jewish General Hospital and Rossy Cancer Network, McGill University, Montreal, QC Canada; 6 Canadian Cancer Trials Group, Kingston, ON Canada; 7 The Ottawa Hospital Cancer Centre, Ottawa, ON Canada; 8 Princess Margaret Cancer Centre and University Health Network, University of Toronto, Toronto, ON Canada

**Keywords:** HER-2, Lapatinib, c-Met inhibitor, Breast cancer, Pharmacokinetics

## Abstract

**Background:**

The mechanisms of resistance to anti-human epidermal growth factor receptor 2 (HER 2) therapies are unclear but may include the tyrosine-protein kinase Met (c-Met), vascular endothelial growth factor (VEGF) and AXL pathways. Foretinib is an inhibitor of c-Met, VEGF receptor 2 (VEGFR-2), platelet-derived growth factor receptor beta (PDGFRB), AXL, Fms-like tyrosine kinase 3 (FLT3), angiopoiten receptor (TIE-2), RET and RON kinases. This phase Ib study sought to establish the associated toxicities, pharmacokinetics (PK) and recommended phase II doses (RP2D) of foretinib and lapatinib in a cohort of HER-2-positive patients with metastatic breast cancer (MBC).

**Methods:**

Women with HER-2 positive MBC, Performance status (PS 0-2), and no limit on number of prior chemotherapies or lines of anti-HER-2 therapies were enrolled. A 3 + 3 dose escalation design was utilized. Four dose levels were intended with starting doses of foretinib 30 mg and lapatinib 750 mg orally once a day (OD) on a 4-weekly cycle. Assessment of c-MET status from the primary archival tissue was performed.

**Results:**

We enrolled 19 patients, all evaluable for toxicity assessment and for response evaluation. Median age was 60 years (34–86 years), 95% were PS 0-1, 53% were estrogen receptor-positive and 95% had at least one prior anti-HER-2-based regimen. The fourth dose level was reached (foretinib 45 mg/lapatinib 1250 mg) with dose-limiting toxicities of grade-3 diarrhea and fatigue. There was only one grade-4 non-hematological toxicity across all dose levels. There were no PK interactions between the agents. A median of two cycles was delivered across the dose levels (range 1–20) with associated progression-free survival of 3.2 months (95% CI 1.61–4.34 months). By immunohistochemical assessment with a specified cutoff, none of the 17 samples tested were classified as positive for c-Met.

**Conclusions:**

The RP2D of the combined foretinib and lapatinib is 45 mg and 1000 mg PO OD, respectively. Limited activity was seen with this combination in a predominantly unselected cohort of HER-2-positive patients with MBC.

**Electronic supplementary material:**

The online version of this article (doi:10.1186/s13058-017-0836-3) contains supplementary material, which is available to authorized users.

## Background

The human epidermal growth factor receptor-2 (HER-2) proto-oncogene is both a prognostic factor and a predictive biomarker for anti-HER-2-targeted therapy. Prior to the advent of trastuzumab, a monoclonal antibody directed against the extra-cellular domain of the HER-2 receptor, the HER-2-positive subtype of breast cancer was associated with a worst prognosis compared to other subtypes of breast cancer [1, 2]. Since the introduction of trastuzumab in the treatment of both early and advanced-stage disease, the prognosis has been significantly altered with improved survival such that this subtype of breast cancer arguably now has one of the better prognoses when treated with anti-HER-2 therapies [3–5].

In the metastatic setting, despite improvements in outcome with HER-2 treatment, progression of disease and death still occur in the vast majority of patients [6, 7]. Treatments designed to avoid or overcome resistance may provide improved outcomes for patients who develop resistance to therapy. Lapatinib is a small molecule tyrosine kinase inhibitor (TKI) of the HER family of receptors - in particular HER-1 and HER-2 [8]. Activity has been demonstrated for lapatinib in combination with hormonal therapy and with chemotherapy in HER-2-positive metastatic breast cancer (MBC) [9, 10]. Despite these demonstrated modest improvements in progression-free survival (PFS), there is either *de novo* resistance to lapatinib or it develops after therapy (acquired resistance). Little is known, however, about these potential mechanisms of resistance.

The tyrosine-protein kinase Met (c-Met) and the vascular endothelial growth factor (VEGF) pathways are proposed mechanisms of resistance to anti-HER-2-targeted therapies. C-Met is a transmembrane receptor tyrosine kinase to which its ligand, hepatocyte growth factor (HGF), binds. Potential oncogenic effects of activation of c-Met include proliferation, angiogenesis, migration and invasion - all hallmarks of a malignant process [11]. Pre-clinical studies have suggested activation of c-Met induces relative resistance to trastuzumab [12]. Likewise, activation of the VEGF pathway is an essential hallmark of cancer and has also been implicated as a resistance pathway to anti-HER-2-directed therapy [13]. Foretinib is an oral tyrosine kinase inhibitor of c-Met, VEGF receptor 2 (VEGFR-2), platelet-derived growth factor beta (PDGFRB), AXL, Fms-like tyrosine kinase 3 (FLT3), angiopoiten receptor (TIE-2), RET and RON kinases, and therefore it is of interest to combine this with anti-HER-2 treatment to overcome putative resistance mechanisms.

The primary objectives of this study were to evaluate the safety profile, and establish the maximum administered dose of foretinib and lapatinib in a cohort of patients with HER-2-positive MBC. Secondary objectives included pharmacokinetics (PK) assessments of each agent, measurement of objective response rates, and evaluation of c-Met in the primary archival tumor specimen.

## Methods

Female patients with HER-2-positive breast cancer (immunohistochemical grade 3+ or fluorescence *in situ* hybridization ratio ≥2.0 as per local assessment), as per American Society of Clinical Oncology/College of American Pathologists (ASCO/CAP) guidelines during the time course of the study, who had incurable loco-regional recurrent or metastatic disease, were enrolled across five Canadian cancer centers. There was no limit on the number of prior systemic therapies (hormonal or chemotherapy) or number of prior anti-HER-2-targeted therapies (including lapatinib) delivered prior to study entry.

Patients were to have a baseline performance status of 0–2, and adequate hematological, hepatic and renal function. Specifically, the eligibility criteria were creatinine ≤1.2 times the upper limit of normal (ULN), total bilirubin ≤1.2 times the ULN and aspartate aminotransferase (AST)/alanine aminotransferase (ALT) ≤2 times the ULN. A baseline left ventricular ejection fraction of ≥50% was also required for study entry. Exclusion criteria included prior exposure to a c-Met inhibitor or to a VEGFR inhibitor; previous history of thromboembolic disease within 6 months prior to study entry; uncontrolled hypertension, active infection, untreated brain metastasis or leptomeningeal disease or serious cardiovascular disease. Measurable disease was not a requirement for the dose escalation phase of the study. Last, tumor specimens were required from all enrolled patients, comprising at minimum a formalin-fixed paraffin-embedded (FFPE) sample from the primary tumor.

This was an open-label multi-center phase I dose escalation study of the combination of foretinib and lapatinib. The study utilized a standard 3 + 3 design with four dose levels. The starting dose of each agent (foretinib 30 mg/lapatinib 750 mg) in dose-level 1 was 50% of each agent given as monotherapy because of the potential for overlapping toxicities. Each agent was given orally (PO) once a day (OD), with 4 weeks duration of therapy defined as one cycle. For the first cycle of each dose level, lapatinib was started on day 1 and foretinib started on day 3. Thereafter they were taken continuously together.

No new patients were entered at an escalated dose level until at least three patients had completed one full treatment cycle at the previous level and the maximum administered dose had not been reached. As an added precaution, the first patient treated at dose-level 1 (DL 1) must have completed one cycle prior to the second and third patient commencing therapy on that first dose level. If no dose-limiting toxicity (DLT) was observed in the first three patients during the first cycle of therapy of each dose level, then the next higher dose level opened to recruitment (Table 1). If one (but not two or more) of the first three patients at a dose level experienced DLT, then an additional three patients were added at that dose level. The maximum administered dose (MAD) was defined as the dose level at which ≥2/6 or ≥2/3 patients experienced dose-limiting toxicity. The next lower dose below the MAD was then declared the recommended phase II dose (RP2D).

**Table 1:** Dose levels of lapatinib and foretinib

Dose level	Foretinib/lapatinib dose	Number of cycles	Number of patients
1	30 mg/750 mg	2	2
		20	1
2	30 mg/1000 mg	2	2
		5	1
3	45 mg/1000 mg	2	2
		3–4	4
4	45 mg/1250 mg	1–2	4
		4	1
		6	2

Adverse events were assessed using Common Terminology Criteria for Adverse Events (CTCAE) version 4.0. DLT was defined as any of the following occurring during cycle 1 of each dose level: (a) grade-3 or worse non-hematologic toxicity (excluding grade-2 alopecia or inadequately controlled/managed diarrhea, nausea/vomiting, rash or hypertension) or hematologic toxicities of grade-4 neutropenia for ≥7 days, grade-3/4 neutropenia with fever, grade-4 thrombocytopenia or anemia or any grade of thrombocytopenia associated with bleeding; (b) the inability to administer cycle-2 treatment within 14 days of the planned dose (i.e. cycle-2 treatment delayed by more than 2 weeks) due to persisting grade-2 drug-related toxicity.

Patients were assessed clinically on day 1 of each cycle with standard hematological and biochemical blood work performed weekly during cycle 1 and then on day 1 of each subsequent cycle. Radiological imaging of the tumor burden was performed at baseline and at the end of every second cycle. Objective response was assessed using Response Evaluation Criteria In Solid Tumors (RECIST) 1.1. Assessment of the left ventricular ejection function (LVEF) was performed following every third cycle of therapy. Limited PK sampling was performed on day 1 and day 29 of cycle 1 only. Patients received treatment until evidence of progressive disease, unacceptable toxicity or elective withdrawal from study.

### C-Met assessment

Archival FFPE material from all consenting study participants was requested for immunohistochemical analysis (IHC) of c-Met using monoclonal antibodies against c-Met (clone SP44; Ventana, Tucson, AZ, USA). The staining was conducted using the Ventana BenchMark Autostainer as per the company's recommended protocol (SP44) as reported previously [14]. Expression levels of c-Met were assessed semi-quantitatively using the standard *H* score ≥200.

### Statistics

This was a phase I study with the primary objective designed to determine the recommended phase II dose of foretinib in combination with lapatinib in patients with recurrent or metastatic HER-2+ breast cancer. The final sample size was dependent upon the number of dose levels required to reach the recommended phase II dose. Once the recommended phase II dose was established, there was an option to enroll up to 15 additional patients to further evaluate toxicity, response and correlative studies of the combination, should an efficacy signal emerge (operationally defined as the observation of at least one objective response) from the dose escalation component of the study.

### Role of the funding source

The study was approved by the local/provincial Research Ethics Board (REB) governing each participating center. All participating subjects signed an informed consent document.

## Results

A total of 19 patients were enrolled across five Canadian cancer centers. The baseline clinical and pathological details of these 19 patients are outlined in Table 2. The majority of patients had performance status 0–1 (95%), had received prior anti-HER-2 therapy for MBC (95%), had received prior chemotherapy (89% including 10/19 who had had three or more prior chemotherapy regimens), and had visceral disease involvement (84%). Of the 19 patients, 8 had been treated both with trastuzumab and lapatinib prior to enrollment into the study.

**Table 2:** Baseline characteristics of the study population (*n* = 19)

Characteristic	Value
Age, years, median (range)	60 (34–86)
Performance status, *n*	
0	9
1	9
2	1
Measurable disease, *n* (%)	16 (84)
Prior therapy, *n*	
Chemotherapy	17
Hormonal therapy	7
HER-2 inhibitor	18
Radiotherapy	15
ER-positive, *n* (%)	10 (53%)
HER-2 positive, *n* (%)	19 (100%)
Number of sites of disease, *n* (%)	
1	5 (26%)
2	2 (11%)
3	6 (32%)
≥4	6 (32%)
Site of disease, *n* (%)	
Bone	9 (47%)
Soft tissue	10 (53%)
Brain	2 (11%)
Lung/liver	16 (84%)

*n* number of patients, *HER*-*2* human epidermal growth factor receptor 2, *ER* estrogen receptor

No DLT was observed during cycle 1 across the first three dose levels (DL 1–3). The third enrolled patient at DL 4 experienced persistent grade-3 fatigue and persistent grade-2 nausea, vomiting, diarrhea and myalgia requiring dose interruption and dose reduction, resulting in a DLT. As a consequence, an additional four subjects were enrolled at DL 4 (foretinib 45 mg/lapatinib 1250 mg). At this expanded dose level, two of these four patients also experienced a DLT in cycle 1. One patient developed grade-3 fatigue and another developed grade-3 diarrhea - both requiring dose reductions. As a consequence, the maximum administered dose was declared at DL 4. Per protocol, a further three patients were enrolled at DL 3. No DLTs were observed in these three additional enrolled patients at this dose level. As such, the RP2D of the combination was deemed to be foretinib 45 mg PO OD and lapatinib 1000 mg PO OD (DL 3).

The most common grade 1–4 adverse events with a frequency ≥20% across DL 3–4 are outlined in Table 3. Most notably, diarrhea, fatigue, elevated liver enzymes, nausea and proteinuria were observed. There was an occurrence of a grade-3 thromboembolic event in two patients and one patient developed both grade-3 proteinuria and grade-3 peripheral edema that was determined to be treatment-related. There was complete resolution of the proteinuria and peripheral edema with discontinuation of foretinib. The spectrum of toxicities was in keeping with target inhibition of c-Met, VEGF, EGFR and HER-2.

**Table 3:** Frequent (≥20%) reported adverse events across dose levels 3–4 that were considered related to both foretinib and lapatinib

	Adverse events Grade 1–2	Grade 3	Grade 4
Dose level 3 (n = 6)			
Diarrhea	6		
Nausea	6		
Vomiting	4	1	
Fatigue	5		
Anorexia	5		
Proteinuria	4		
Thromboembolic	1	2	
Anemia	4		
AST and/or ALT elevation	5/6		
Dose level 4 (n = 7)			
Diarrhea	2	5	
Nausea	7		
Vomiting	5		
Fatigue	5	2	
Anorexia	5		
Proteinuria	2	1	
Edema (limbs)	2		
Myalgia	3		
Hypertension	4		
Anemia	2		
AST and/or ALT elevation	5/4	2/3	

*AST* aspartate aminotransferase, *ALT* alanine aminotransferase

Limited sampling for PK was undertaken in this study. The PK data of the drugs in combination was similar to historical data on the use of the drugs as single agents, suggesting no significant apparent interactions (Additional file 1: Table S1A and Table S1B).

**Fig. 1:**
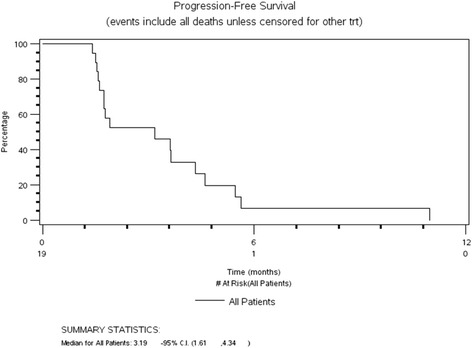
Progression-free survival (*PFS*). *trt* treatment

**Fig. 2:**
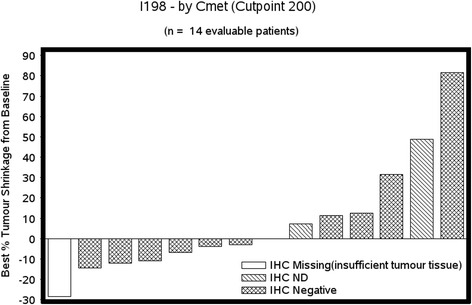
Best tumor shrinkage based on tyrosine-protein kinase Met expression. *IHC* immunohistochemial analysis

Among the 19 enrolled patients, there were no documented responses according to the RECIST criteria. In 6 patients (32%) the best response was stable disease (median duration 4.3 months, range 3.0–10.9 months) and in 12 patients it was progressive disease; response could not be evaluated in 1 patient. The median PFS in the study cohort was 3.2 months (95% CI 1.61–4.34 months) (Fig. 1). Among the 19 patients enrolled, c-Met was evaluated by IHC in 14 patients (there were no results for 5 patients, 4 patients had no target lesions and response was not evaluable in 1 patient). With the predefined cut point ≥200 (considered positive for c-Met), none of the patients had tumors positive for c-Met, 15 patients had tumors that were negative for c-Met and in 4 patients the status was unknown or not assessable (due to insufficient tumor tissue from 2 patients, and to status not being assessed in the other 2 patients because the tissue had been returned to the original pathology laboratory). The best tumor shrinkage and c-Met status are summarized in a waterfall plot (Fig. 2).

## Discussion

Despite the initial dramatic benefits seen with adjuvant trastuzumab concurrent or sequential to chemotherapy, relapses are unfortunately still occurring over time. At the first analysis of the pivotal NSABP B-31 and NCCTG N9831, with 2 years of median follow up, the disease free survival (DFS) was 87.1% [14]. Now, with longer follow up, the rate of DFS at year 10 was 73.7% [15].

In advanced-stage disease, despite significant improvements in PFS and overall survival with dual HER-2 antibody blockade (pertuzumab and trastuzumab) and with an antibody drug conjugate (TDM1), progression of disease and death ultimately occurs in the vast majority of patients [6, 7, 16]. The CLEOPATRA trial demonstrated the combination of pertuzumab, trastuzumab and docetaxel was superior to trastuzumab and docetaxel in both PFS (median PFS 18.7 months vs 12.4 months) and overall survival (OS) (median OS 56.5 months vs 40.8 months), respectively [6, 16]. The EMELIA trial demonstrated that the antibody drug conjugate (TDM1) was superior to the combination of lapatinib and capecitabine in both PFS (median PFS 9.6 months vs 6.4 months) and OS (median OS 30.9 months vs 25.1 months) respectively [7]. Though these two landmark trials have established the preferred treatment algorithm in first-line and second-line disease, additional therapeutic agents are still needed in the management of HER-2-positive MBC, particularly those that may circumvent or overcome resistance to targeted HER-2 therapy.

The combination of TDM1 and pertuzumab was studied in the phase III MARIANNE trial comparing it to TDM1 alone or docetaxel and trastuzumab as first-line therapy for HER-2-positive MBC, unfortunately with no improvements in PFS demonstrated for the TDM1 and pertuzumab combination arm relative to the other arms investigated [17]. As mutations in the phosphatidylinositol 3 kinase (*PIK3CA*) exons 9 and 20 gene and/or loss of the tumor suppressor gene *PTEN* occurs frequently in breast cancer with over-expression of HER-2 and is associated with a poorer outcome and relative trastuzumab resistance, investigations into blocking this pathway in combination with trastuzumab have been investigated. Everolimus is a mammalian target of rapamycin (mTOR) inhibitor with demonstrated clinical activity in combination with hormonal therapy in ER+ MBC [18]. The BOLERO-1 investigated the combination of everolimus with paclitaxel and trastuzumab vs placebo with paclitaxel and trastuzumab as first-line therapy in HER-2-positive MBC. The BOLERO-3 investigated the combination of everolimus with vinorelbine and trastuzumab vs placebo with vinorelbine and trastuzumab as second-line therapy or later in HER-2-positive MBC. In a combined translational study there was correlation between greater benefit and everolimus in the cohort with PIK3CA mutation, with a hazard ratio of 0.67 (95% CI 1.45–1.0) [19]. Though supporting the hypothesis for current clinical trials of PIK3CA inhibitors in combination with anti-HER-2 therapy, the clinical utility of evolimus and trastuzumab with chemotherapy is limited by their toxicities and the changing landscape of HER-2 agents.

Lapatinib is a small molecule tyrosine kinase inhibitor of HER-1 and HER-2. The proposed mechanism of anti-tumor activity is the inhibition of the downstream pathways of PI3K/Akt and mitogen-activated protein kinase (MAPK), induction of apoptosis and inhibition of proliferation, which results from inhibiting phosphorylation of these receptors by lapatinib [20]. Lapatinib in combination with capecitabine in metastatic HER-2-positive breast cancer following prior trastuzumab exposure has demonstrated improved PFS to 8.4 months for the combination vs 4.4 months for capecitabine alone [10]. However, no overall survival difference between the regimens was seen, likely in part due to cross-over after the study was halted. Inconsistent results have been demonstrated for the addition of lapatinib to hormonal therapy in hormone-receptor-positive HER-2-positive MBC, with some improvement in PFS in combination with letrozole, but no benefit seen in combination with fulvestrant [9, 21]. Last, the combination of lapatinib and trastuzumab demonstrated superior efficacy compared to lapatinib alone in a trial of HER-2-positive MBC that was refractory to trastuzumab [22].

Mechanisms of resistance to anti-HER-2 therapies, in particular the small molecule TKIs are widely speculated but not validated in clinical practice. Broadly, the main mechanisms of resistance to lapatinib can be categorized into activation of compensatory pathways, mutations of the HER-2 tyrosine kinase (TK) domain, and gene amplification. Within the category of activation of compensatory pathways, the c-Met and AXL receptor tyrosine kinases (RTKs) have been implicated as potential key mediators. The c-Met TK is a transmembrane receptor for hepatocyte growth factor (HGF), which upon activation is associated with increased proliferation, cell invasion and evasion of apoptosis [23, 24]. Expression of c-Met has been associated with resistance to both trastuzumab and lapatinib [25]. More importantly, in a pre-clinical study of the combination of a c-Met TKI and lapatinib in an HER-2-positive gastric cell line, resistance to lapatinib was overcome with restoration of growth inhibition [26].

AXL is a transmembrane receptor with an intracellular kinase domain that has structural similarities to c-Met. Mechanistically, activation of the pathway engages the PI3K regulatory subunit p85 to activate the PI3K pathway, and by doing so bypasses upstream blockade. Activation of the pathway has been associated with clinical resistance to TKIs such as imatinib for gastrointestinal stromal tumors (GIST), epidermal growth factor receptor (EGFR) TKIs in non-small cell lung cancer and to lapatinib in pre-clinical breast cancer models [26, 27]. A multi-kinase AXL inhibitor has demonstrated restoration of sensitivity to both lapatinib and trastuzumab in a breast cancer model with acquired resistance to lapatinib [28].

A phase II study of single-agent foretinib in advanced-stage triple-negative breast cancer (TNBC) has been performed and recently published by our group [29]. A daily dose of foretinib of 60 mg PO was delivered in 45 patients (37 evaluable) with the most common grade-3 or greater toxicities seen as hypertension (49%), diarrhea (7%), nausea (4%) and fatigue (4%). Though the response rate was low (4.7%), a stable disease rate of 33% was demonstrated (median duration 5.4 months). There was no correlation between Met status of the tissue or in circulating tumor cells (CTC) and clinical activity.

To our knowledge, this is the first published clinical study of a combination of an anti-HER-2 agent with a c-Met inhibitor in breast cancer. Overall the toxicity profile was in keeping with known toxicities of both agents, with the most common grade 3–4 adverse events occurring at the maximal administered dose (DL 4), and consisting of fatigue and diarrhea. Other prominent grade-2 toxicities at this higher dose level were anorexia, nausea, vomiting and musculoskeletal symptoms. Two subjects developed thromboembolic disease, and c-Met inhibitors are known to increase the rate of thrombosis [30, 31]. In the entire study population, however, we did not demonstrate any responses and the median PFS of 3.2 months was disappointing. Though we had intended an exploratory analysis of response or clinical benefit in c-Met-positive tumors, unfortunately none of the samples that were collected within the study population were classified as c-Met-positive by IHC and by the defined staining cutoff.

Beyond the limited efficacy in this study, other limitations of our study included a relatively small sample size at the RP2D (n = 6), lack of assessment of c-Met or other biomarkers on a more contemporary tumor specimen reflective of the metastatic phenotype, lack of pharmacodynamic modulation assessment for the combination of drugs, and the broad inclusion of any HER-2-positive disease. The absence of demonstration of c-Met expression in our population also may cast doubt on the hypothesis of this being an important target for resistance modulation to anti-HER-2 therapy.

## Conclusions

In our phase-I study, we have determined the recommended doses for consideration in future studies of the combination of foretinib and lapatinib to be 45 mg and 1000 mg PO daily, respectively. At our recommended phase-II dose level, the combination appears relatively tolerable apart from modest risks of diarrhea, fatigue, anorexia and nausea. There was limited efficacy (median PFS 3.2 months (95% CI 1.61–4.34 months)) in our study population. Future studies of this combination, if considered, should likely only include a population that demonstrates activity of either the c-Met and/or AXL pathways as drivers of either primary or acquired resistance to anti-HER-2-directed therapies.

## Additional file

Additional file 1: Table S1A. Pharmacokinetics of lapatinib. **Table S1B.** Pharmacokinetics of foretinib. (DOCX 558 kb)

## Abbreviations

ALT: Alanine aminotransferase
AST: Aspartate aminotransferase
CCTG: Canadian Clinical Trials Group
c-Met: Tyrosine-protein kinase Met
DL: Dose level
DLT: Dose-limiting toxicity
EGFR: Epidermal growth factor receptor
FFPE: Formalin-fixed paraffin-embedded
FLT3: Fms-like tyrosine kinase 3
HER-2: Human epidermal growth factor 2
HGF: Hepatocyte growth factor
MAD: Maximal administered dose
MBC: Metastatic breast cancer
mTOR: Mammalian target of rapamycin
OS: Overall survival
PDGRFB: Platelet-derived growth factor receptor beta
PFS: Progression-free survival
PI3K: Phosphatidylinositol 3 kinase
PK: Pharmacokinetics
PO: Per oral
PTEN: Phosphatase and tensin homolog
RP2D: Recommended phase II dose
TK: tyrosine kinase
TKI: Tyrosine kinase inhibitor
VEGF: Vascular endothelial growth factor
