# Supplementary material for: A phase-I study of lapatinib in combination with foretinib, a c-MET, AXL and vascular endothelial growth factor receptor inhibitor, in human epidermal growth factor receptor 2 (HER-2)-positive metastatic breast cancer
Source: Breast Cancer Res. 2017 May 2;19:54. doi: 10.1186/s13058-017-0836-3 (PMC5414192; doi:10.1186/s13058-017-0836-3)
Supplement: Additional file 1: Table S1A. — Pharmacokinetics of lapatinib. Table S1B. Pharmacokinetics of foretinib. (DOCX 558 kb) [file 13058_2017_836_MOESM1_ESM.docx]

Table S1A – Pharmacokinetics of lapatinib

**** Table S1B – Pharmacokinetics of foretinib
